# Comparative mechanical characterisation of 13–93 bioactive glass and hybrid scaffolds for bone regeneration

**DOI:** 10.1038/s41598-026-46620-9

**Published:** 2026-04-03

**Authors:** Jingwen Liu, Jishizhan Chen, Agathe Heyraud, Aikta Sharma, Meryem Lamari, Francesca Tallia, Julian R. Jones, Peter D. Lee

**Affiliations:** 1 Mechanical Engineering, University College London, Torrington Place, London, WC1E 7JE UK; 2 Department of Materials, Imperial College, South Kensington, London, SW7 2AZ UK

**Keywords:** Biomaterial, Bone regeneration, µCT, Mechanical characterisation, Bioactive glass, Biotechnology, Engineering, Materials science

## Abstract

Biomaterial bone scaffolds offer a promising alternative to traditional treatments. Bioactive glass has shown promise however its clinical application is limited by its poor mechanical properties. Flexible hybrids are a potential solution. Herein, previous studies incorporated calcium into a silica/poly(tetrahydrofuran)/poly(ε-caprolactone) (SiO₂/PTHF/PCL-diCOOH) sol-gel hybrid to enhance its mechanical performance. We firstly compared these material scaffolds to 13–93 bioactive glass (13–93 BG) (54.6% SiO_2_, 22.1% CaO, 6.0% Na_2_O, 7.7% MgO, 7.9% K_2_O, and 1.7% P_2_O_5_, in mol%) scaffolds. We reveal that the hybrid material exhibits a randomised porous microstructure and superior mechanical deformability, whereas the 13–93 BG scaffolds remain stiffer and more brittle. Micro-computed tomography (µCT) quantification reveals that the glass scaffolds underwent less shrinkage after direct ink writing (DIW) than the hybrid scaffolds, enabling easier reproduction of the design files. We demonstrate that the hybrid scaffold can withstand strains of up to 7%, mimicking the elastic behaviour of bone, while the 13–93 BG scaffold fails at 2% strain. Finite element (FE) analysis revealed a more decentralized stress distribution and higher local strain within the hybrid scaffold, whereas the 13–93 BG scaffold showed high stress concentration at strut junctions in line with a higher risk of brittle failure. The characterisation methods applied in this study can be extended to other biomaterials, providing valuable insights for biomaterials research and guiding scaffold design for bone regeneration.

**Supplementary Information:**

The online version contains supplementary material available at 10.1038/s41598-026-46620-9.

## Introduction

Bone defects can result from trauma, infections, tumours or pathological conditions such as osteoporosis, all of which may compromise bone structure and integrity^1,2^. Addressing these defects remains a significant clinical and research challenge, particularly in treating large bone defects (4–5 cm in length or greater)^3^ where non-union healing processes are indicative of failure to heal or respond to standard therapies^4^. Currently, methods for repairing bone defects autologous bone grafts are considered the gold standard due to their osteogenic potential. An autologous bone graft refers to a surgical procedure in which bone tissue is harvested from one part of a patient’s body (the donor site) and transplanted to another site in the same individual to repair or regenerate bone^5^. However, their use is limited by donor site morbidity and availability^6^.

Bioactive glass forms a biological bond with host tissue after implantation^7^. Due to its osteoconductive and bioactive properties, bioactive glass has significant potential for repairing damaged or diseased bone^8^. Massera et al. investigated the surface functionalization of phosphate-based bioactive glasses using 3-aminopropyltriethoxysilane (APTS) to enhance their bioactivity and cell compatibility. The study demonstrates that APTS modification improves the biological performance of phosphate-based bioactive glasses for potential tissue engineering applications^9^. Commercially, it is available in the form of particulates or granules only. Since the development of the first clinically applied bioactive glass compositions such as 45S5 Bioglass^®^, commercial bioactive glasses have been widely used in orthopaedics, dentistry, and bone regeneration^10^ due to their proven biocompatibility, osteoconductivity, and ability to form a strong bond with host bone through surface apatite formation.

Biomaterial bone scaffolds offer distinct advantages over granules in bone repair^11^ as they can act as three-dimensional (3D) templates for tissue growth, including vascularised bone ingrowth, and allow the optimization of mechanical properties, degradation rates, and biocompatibility^2^. Additive manufacturing (AM) permits the construction of intricate structures layer by layer from 3D model data^12^. Recent advances in additive manufacturing have enabled the fabrication of highly tailored bioactive glass and composite scaffolds for bone tissue engineering. For example, Baino and Fiume^13^ demonstrated the 3D printing of hierarchical mesoporous bioactive glass scaffolds with controlled macro- and micro-porosity for enhanced functionality. Likewise, Boccaccini’s group has developed polymer–bioactive glass composite scaffolds via fused deposition modelling, which demonstrated good printability, cytocompatibility, and enhanced osteogenic differentiation compared to pure polymer scaffolds^14^.

Direct ink writing (DIW) involves extruding ink-like materials through a nozzle to create detailed 3D structures with fine resolution. In general, DIW can be used in two ways to print materials: (i) printing the materials directly; and (ii) particulates can be printed in a binder that can be burned out during sintering processes^15^. In both cases, the “ink” must exhibit shear thinning rheology. For metals, ceramics and glasses the binder is removed during calcination, and the particles must sinter efficiently. The loading of the particles in the binder must be maximised.

DIW has been widely used to fabricate titanium scaffolds that exhibit stiffness and yield strength comparable to cancellous bone^16^. For bioactive glasses, a sintering temperature must be chosen that lies between the glass transition temperature and crystallisation onset temperature of the glass. For commercial bioactive glasses, this sintering window is too narrow, so other compositions must be selected, such as ICIE 16 (49.46% SiO_2_, 36.27% CaO, 6.6% Na_2_O, 1.07% P_2_O_5_ and 6.6% K_2_O, in mol%) and 13–93 BG (54.6% SiO_2_, 22.1% CaO, 6.0% Na_2_O, 7.7% MgO, 7.9% K_2_O, and 1.7% P_2_O_5_, in mol%)^15^. Bioactive glass scaffolds fabricated using DIW have shown good mechanical strength during in vitro degradation^17^. However, DIW often requires post-processing steps (drying and sintering) and confinement of the printing process to the *x*–*y* plane restricts the fabrication of complex 3D architectures^18^.

Bioactive glass scaffolds can mimic the porous structure of trabecular bone and achieve comparable compressive strengths, but the inherent brittleness of glass limits their use, as they cannot withstand cyclic loads^19,20^. Incorporating bioactive glass into a polymer matrix can address its low brittleness by forming a composite with improved mechanical properties. However, the extent of bone formation can be reduced by the surface of the glass being masked by the polymer matrix. The different degradation rates of the polymer in comparison to bioactive glass also result in scaffold instability^19^. To overcome these limitations, a biodegradable polymer can be incorporated into the inorganic bioactive glass at the molecular level, forming a hybrid material. An example is the SiO_2_/PTHF/PCL-diCOOH sol-gel hybrid material, synthesised by Tallia et al.^21^, which exhibits exceptional mechanical properties, including elastic ‘bounciness’ and intrinsic autonomous self-healing. 3D-printed SiO_2_/PTHF/PCL-diCOOH scaffolds with 200 µm pores promoted human bone marrow stem cells to differentiate into chondrocytes, which expressed articular cartilage-specific markers in vitro^21,22^.

The inorganic component of these hybrid scaffolds was only silica. Adapting the SiO_2_/PTHF/PCL-diCOOH scaffolds for bone regeneration required introducing calcium into the silicate network so that it became a closer mimic of the original bioactive glass, but the introduction of calcium was not trivial as new calcium sources were needed. Calcium methoxyethoxide (CME) was used as a calcium precursor by Tallia et al.^23^, to produce SiO_2_-CaO/PTHF/PCL-diCOOH hybrids and DIW was used to create an interconnected porous bone scaffold. Heyraud et al. investigated different calcium sources to enhance the printability of hybrid inks and the mechanical properties of scaffolds^24^. Based on printability, degradability, and mechanical analysis, scaffolds fabricated using calcium ethoxyethoxide (CEE) demonstrated superior material printability and improved microstructural integrity. By optimizing the inorganic composition to a 70:30 molar ratio of tetraethylothosilicate (TEOS) to CEE, the scaffold properties were further refined^25^. This optimization involved increasing the strut size while preserving the interconnected channel network, resulting in optimal failure stress and toughness modulus. The identified composition was determined to be the most effective for bone regeneration applications.

Prior studies have demonstrated the potential of both 13–93 BG and hybrid scaffolds, but these materials have so far been studied separately. Therefore, it has remained difficult to distinguish which differences in scaffold performance arise from the material class itself and which reflect variations in scaffold design or testing conditions. The key novelty of this work is that we have decoupledthe effects of material composition and scaffold architecture. This study aims to present a critical comparison of SiO_2_-CaO/PTHF/PCL-diCOOH hybrid and 13–93 BG material for bone regeneration. Although the two systems differ in chemical composition, both are designed to support bone regeneration under load-bearing conditions. More importantly, this comparison is not intended to directly compare intrinsic material properties, but rather to evaluate their performance within a controlled, application-relevant framework. The same manufacturing route, comparable scaffold architectures, and identical mechanical testing protocols ensure that key variables are fixed, enabling material-dependent effects on microstructure and mechanical response. This provides a meaningful basis for comparison under equivalent structural and loading conditions. The comparison encompasses a comprehensive analysis of their material properties, 3D printability, microstructural characteristics, and mechanical performance. The findings of this study are expected to guide the development of these two advanced biomaterials for clinical applications in bone repair and regeneration, contributing to the broader field of tissue engineering and regenerative medicine.

## Materials and methods

## 13–93 bioactive glass synthesis

The 13–93 BG used in this study was produced via a melt-derived quenching method. The glass was formulated to match the 13–93 composition: 54.6% SiO_2_, 22.1% CaO, 6.0% Na_2_O, 7.7% MgO, 7.9% K_2_O, and 1.7% P_2_O_5_, in mol%. High purity silica sand was purchased from Prince Minerals, Stoke-on-Trent, UK). All other reagents were purchased from Sigma-Aldrich UK with a minimum purity of 96%, unless otherwise stated. The powdered reagents were thoroughly mixed and melted in a Pt–5%Au crucible at 1400 °C for 1.5 h in an elevator hearth furnace (EHF 17/3, Lenton^®^). The molten glass was then quenched by pouring it into distilled water at room temperature^26^. The reduced thermal gradient between the molten glass and quenching medium minimized thermal shock, thereby suppressing crack formation in the glass frit. The resulting glass frit was dried, milled, and sieved to obtain particles smaller than 32 μm. The amorphous structure of the 13–93 BG was confirmed by X-ray diffraction (XRD). Subsequently, the chemical composition was verified through inductively coupled plasma optical emission spectroscopy (ICP-OES) following sample acid digestion.

## 3D printing of 13–93 bioactive glass material

Inks for 3D printing were made with 45 wt% glass in a solution of 55 wt% Pluronic F-127. Pluronic was first dispersed and mixed in deionised water, then stored in a refrigerator for 2 min to allow it to partially liquefy. After removal from the refrigerator, the solution acquired a gel-like consistency, which was further broken down and homogenised by continuous stirring until the Pluronic was fully dissolved. The prepared Pluronic solution was then stored at 5 °C. 45 wt% of 13–93 BG frit was gradually added to the Pluronic solution in multiple steps to aid dispersion. Once a uniform and pasty slurry was obtained, the mixture was further homogenised using a Thinky mixer at 2200 rpm for 5 min. The final ink was stored in a refrigerator at 5 °C to maintain its printability. Prior to robocasting, the ink was transferred into 3 mL syringes approximately one hour in advance of printing, which was found to be the optimal conditioning time to ensure stable and continuous flow during printing.

The 13–93 BG inks were printed using a Robocaster with the RoboCAD software (3d Inks LLC, USA). The strut size was determined by the nozzle used, whereas the strut separation and layer thickness were determined by the design file. A speed of 10 mm s^−1^ and deposition rate of 0.08 mL min^−1^ were used. This deposition rate was held constant during printing by the automatic force adjustment applied to the plunger on the z-axis. Strut spacing of 650 μm ensured a final pore size of 400–500 μm post sintering. The layer thickness was set to 332 μm, due to the properties of the 13–93 BG inks, the layer thickness was set to the is the tip size (400 μm) divided by 1.2 to account for some collapsing of struts when deposited. The cylinder was built with a radius of 5.15 mm and 42 layers or a height of 13.994 mm to ensure a final diameter and height of 8 mm and 12 mm post sintering. To sinter the 13–93 BG scaffolds, they were first left to dry overnight at RT, and then placed in a furnace with an initial ramp to 550 °C at 3 °C min^−1^, followed by a 1 h dwell, another ramp to 690 °C at 3 °C min^−1^ and a final dwell of 1.5 h.

## Hybrid material synthesis

CEE was synthesized by heating and stirring 48 mL of 2-ethoxyethanol at 80 °C in a paraffin bath at 600 rpm in an argon environment to prevent oxidation^27,28^. 2 g of calcium was then added to the heated flask, prior to re-purging with argon until the temperature reached and was maintained at 125 °C for 20 h. Following this, the yield was centrifuged at 6000 rpm for 20 min to allow density-mediated separation and collection of an inorganic transparent red solution, which was the final CEE solution.

In the inorganic sol, TEOS and CEE solution were homogenized at a 70:30 molar ratio to achieve better printability and mechanical properties and stirred at 800 rpm for three hours^29^. Simultaneously, PCL-diCOOH (poly(ε-caprolactone)) (1 mol), (3-glycidyloxypropyl) trimethoxysilane (GPTMS, 2 mol), and boron trifluoride diethyl etherate (BF₃·OEt₂, 0.5 mol) were reacted in tetrahydrofuran (THF), which was used at a PCL-diCOOH to THF ratio of 100 mg/mL, at 400 rpm for 1.5 h to form the organic solution. A 70:30 wt% TEOS: PCL ratio was used to generate the hybrid material. After the reactions in the inorganic and organic solutions were completed, the organic solution was then added dropwise to the inorganic solution under continuous stirring at 400 rpm and room temperature over a period of 1.5 h. Deionized water was then introduced to hydrolyse TEOS and GPTMS, followed by the addition of 2 M nitric acid. A TEOS to deionized water of 1:2 mol% was maintained to prevent rapid gelation and avoid premature calcium reactions with water before the hydrolysis of TEOS and GPTMS. Nitric acid (2 M) was added at a 1:6 v/v ratio relative to the volume of water. Fourier transform infrared (FTIR) spectroscopy was employed to verify the functional groups of the organic and inorganic components and to investigate the consequent variations in the hybrid’s chemical structure and bonding with calcium additions^30^.

## Hybrid material moulding of bulk samples

To create bulk samples from the hybrid material, the hybrid sol was transferred into cylindrical PTFE molds (7 mm in thickness) and sealed within polymethylpentene (PMP) containers for 3 days before gelation occurred. To prevent rapid drying and cracking, 1.5–2 mL of 70% aqueous ethanol was added to the PMP containers to facilitate slow and controlled drying. Gradual drying was achieved by loosening the container lids, first at 40 °C for approximately 2 weeks (until the ethanol completely evaporated) before the product was dried in a 60 °C oven for approximately one week. The density of the hybrid calcium material was determined in air and water using a Density Determination Kit (OHAUS, Switzerland) with water as the auxiliary liquid.

## 3D printing of hybrid material

Based on the protocol established by Heyaurd et al.^25^, 3D-printable hybrid inks were synthesized. The printing parameters were subsequently optimized for this study. To prepare the hybrid material ink, the final solution after the addition of water and acid was stirred for an additional 10 min to induce the onset of gelation. The sol was transferred into 3 mL syringes and stored in a freezer at − 82 °C. To achieve controlled strut thickness and pore size of the scaffolds, DIW (Robocaster, 3d Inks LLC, USA), was used to print hybrid scaffolds. The syringe containing the hybrid calcium material ink was brought from the freezer to room temperature for around 30 min to allow the ink to defrost for printing. By applying pressure to the plunger, the ink was extruded through the nozzle. To obtain a scaffold with a diameter of 8 mm and a height of 12 mm, a bespoke computer-aided design model with dimensions of 14 × 14 mm and a height of 16.8 mm was utilized. The strut thickness of 400 μm, the same as that for the 13–93 BG scaffolds, determined by the diameter of the syringe nozzle, strut separation of 1.25 mm, pore size of 0.85 mm and layer separation of 0.4 mm were achieved using a printing speed of 10 mm/s and deposition rate of 0.175 mL/min. After printing, the scaffolds were aged at 40 °C for 3 days in airtight sealed poly (methyl pentene) (PMP) pots, followed by drying for 10 days at 40 °C. During this period, the pots were gradually opened by a ¼ turn each day until fully loosened, resulting in approximately 30% shrinkage of the scaffolds. After aging and drying, the scaffolds were cut to the desired size using an 8 mm punch.

## Micro-computed tomography imaging of 13–93 bioactive glass and hybrid scaffolds

To investigate the microstructure of 13–93 BG and hybrid scaffolds, a commercial µCT machine (Nikon XTH225 ST, UK) was utilized. Scaffolds were mounted on the sample stage using a double-sided tape in their native state. Scans were performed with a voxel resolution of 7 μm, under a beam current of 140 µA and a voltage of 70 kV to provide detailed insights into the internal structure of the scaffolds. The µCT images were reconstructed using Nikon’s CT Pro 3D software (XT 6.12.1, UK), and post-processed using a 3 × 3 × 3 median filter to reduce noise in the images. Manual thresholding was then performed in Avizo (version 2023), whereby the midpoint between attenuation peaks weas selected in preparation for image segmentation. Following segmentation, the volume and surface area of the bone scaffolds were measured using the ‘Label Analysis’ tool in Avizo. Open porosity represents the volume fraction of interconnected pores within the total pore volume of the scaffold, indicating the degree of pore connectivity in the structure. To quantify total and open porosity, a cylindrical sub-volume encompassing 95% of the scaffold was selected for each sample. Using the ‘Label Analysis’ module, the volumes of all pores and the subset of connected pores within the sub-volume were calculated. The permeability of each scaffold was measured using ‘Absolute Permeability Experiment Simulation’ module in Avizo^31^. For 3D channel size and strut thickness, the measurements were conducted on the cylindrical sub-volume of scaffolds using ImageJ, an open-source image processing program, in combination with the BoneJ plugin^32^. After acquiring the channel sizes and strut thickness values, visualization of the 3D distributions was performed in Avizo.

## Mechanical testing

Uniaxial mechanical compression testing was conducted on hybrid bulk samples and 3D printed 13–93 BG and hybrid bone scaffolds using a machine with a 5000 N load cell (Instron, Norwood, USA). Mechanical tests were conducted at a constant speed of 2.5 mm/min under ambient laboratory conditions. The height and diameter of each bulk sample and scaffold were precisely measured using a digital calliper with an accuracy of ± 0.01 mm. During testing, force and displacement data were continuously recorded in Bluehill^®^ Software. Engineering stress was calculated as force divided by the initial cross-sectional area, and engineering strain as displacement divided by the original height. True stress and strain were derived from engineering values by accounting for the continuous change in specimen dimensions during deformation and used to generate stress-strain curves, enabling a comparison of mechanical performance between the two material samples.

Cyclic compressive testing was conducted on hybrid scaffolds using an Instron 5000 Universal Testing Machine (Instron, USA). As the 13–93 BG scaffolds were inherently brittle, cyclic compressive testing was difficult to perform. Tests were performed under force control at a constant loading speed of 5 mm/min, with each sample subjected to 10 loading-unloading cycles up to a maximum compressive force of 50 N. Force and displacement data were continuously recorded throughout the cycles. The resulting data were used to generate stress-strain curves as above providing insights into the cyclic mechanical behaviour and structural resilience of the hybrid calcium scaffolds.

## Finite element modelling of scaffolds under uniaxial compression

FE simulations were conducted using ABAQUS/Standard (v11, Simulia, Providence, RI). Models of 13–93 BG and hybrid scaffolds were generated from µCT images in Avizo (v2023, Thermo Fisher Scientific). Top and bottom surfaces were flattened and scaffold heights were standardized to 9 mm. To simulate loading, a vertical displacement was applied to the top surface nodes while the bottom surface was fully constrained. Lateral degrees of freedom on the top nodes were left unconstrained to represent frictionless, conformal contact without explicit platen modelling. This boundary condition strategy was adopted to prioritize the analysis of internal stress and strain distributions within the scaffold microstructure over replicating exact experimental load-displacement behaviour. Given the scaffold height, the influence of surface flattening and these boundary simplifications is expected to be minimal relative to the loaded regions. A mesh convergence study was carried out by varying the minimum element size. The mesh size with the best balance between accuracy and computational efficiency was selected for simulations. Optimised meshing was performed in Avizo with tetrahedral elements. Material properties including elastic modulus and density were assigned based on experimental measurements (Table S1). A Poisson’s ratio of 0.3 was applied to the hybrid models and a value of 0.25 was used for the 13–93 BG models, consistent with silicate glass properties^33^. Compression was simulated using displacement-controlled boundary conditions with vertical displacements of 0.45 mm, 0.90 mm, and 1.35 mm applied to the top surface, corresponding to nominal compressive strains of 0.5%, 1.0%, and 1.5%, respectively, relative to the standardized scaffold height of 9 mm. The FE analysis was intended to compare the internal micromechanical stress/strain distribution and localisation patterns between scaffold types under small-strain elastic loading, rather than to reproduce the full experimental stress-strain response or quantitatively predict failure.

## Statistical analysis

Data were analyzed using two-way ANOVA, followed by Tukey’s test for pairwise group comparisons. Five hybrid bulk samples, six 13–93 BG and hybrid scaffold samples were included in each test and analysis. Statistical significance was determined using the Mann-Whitney U test: * indicates *P* < 0.05, ** indicates *P* < 0.01, *** indicates *P* < 0.001 and **** indicates *P* < 0.0001. All analyses were performed using GraphPad Prism version 10 (GraphPad Software, USA).

## Results and discussions

### Bulk material properties

The hybrid bulk samples exhibit an orange translucent appearance (Fig. S1a) and a cylindrical shape with a mean diameter of 8.3 mm and a height of 6.9 mm. The density of the hybrid material was 1370 kg/m^3^, significantly lower than the reported density of bulk 13–93 BG, which ranges from 2440 kg/m^3^ to 2880 kg/m^3^^34–36^. Under compression testing, the stress–strain curves of the hybrid bulk samples typically show an initial linear increase in true stress with true strain which corresponds to the elastic deformation region (Fig. S1b)). Pauses in this linear increase could be caused by defects, bubbles or cracks within the bulk samples, which reduce local strength and can lead to collapse or fracture resulting in stress fluctuations. The average elastic modulus of the hybrid bulk samples was 139 ± 16 MPa and the average modulus of toughness was 1395 ± 550 kJ/m^3^, reflecting the material’s ability to absorb energy before fracture. According to the literature, the elastic modulus of 13–93 BG is 86 GPa, which is much higher than the hybrid (139 ± 16 MPa)^35^. Similarly, the compressive strength of 13–93 BG (69 MPa) is much higher than that of the hybrid (18 ± 4 MPa), indicating its capability to withstand markedly higher compressive loads (Table S1). These values suggest that 13–93 BG exhibits superior mechanical properties compared to the hybrid. However, there is limited research available on the mechanical performance of pure bioactive glass in its solid form but most studies focus on its use as a scaffold^37,38^.

## Direct ink writing of 13–93 bioactive glass and hybrid scaffolds

**Fig. 1:**
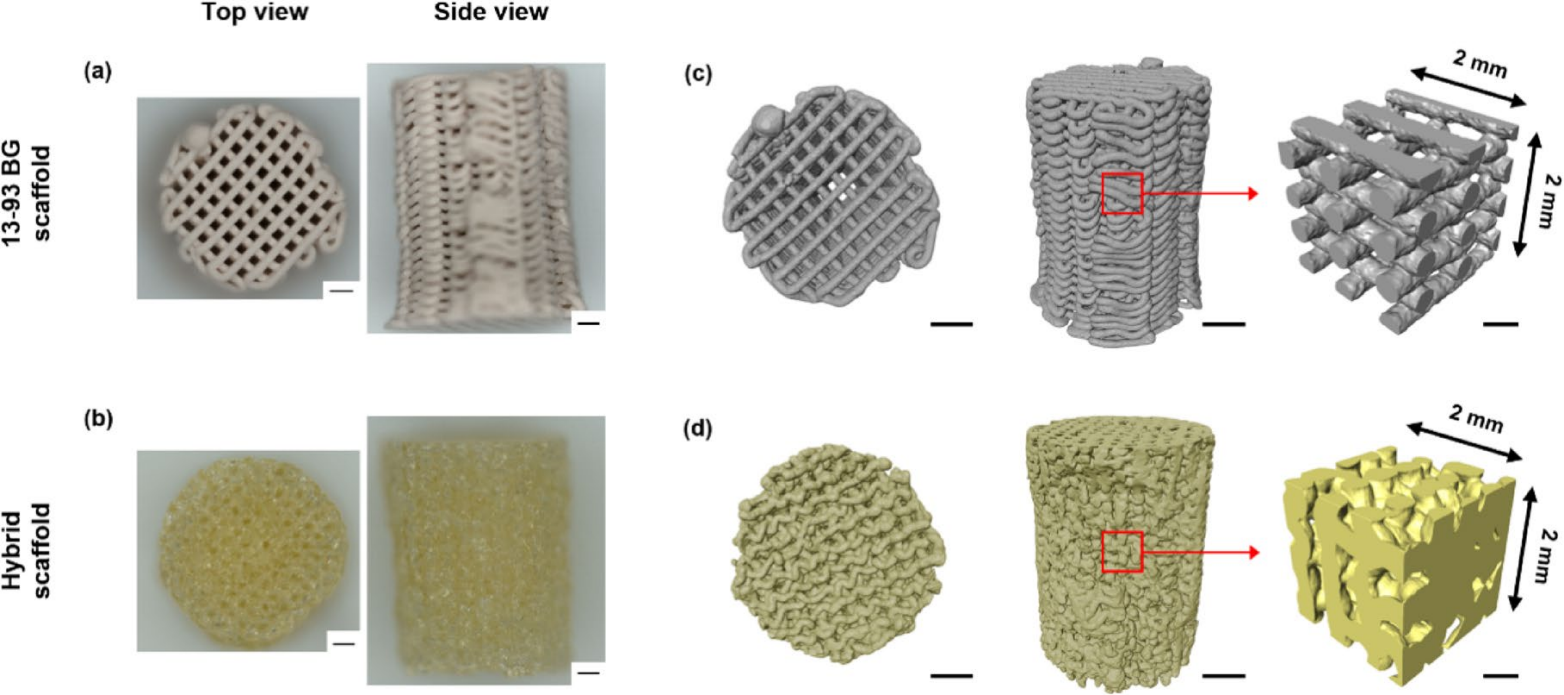
3D-printed samples and µCT imaging of scaffolds of hybrid and 13–93 BG: 3D-printed (**a**) 13–93 BG and (**b**) hybrid scaffold (scale bar: 1 mm); (**c**, **d**) µCT 3D rendering showing top and side views of the 13–93 BG and hybrid scaffold (scale bar: 2 mm) and selected VOIs (2 × 2 × 2 mm, scale bar: 0.5 mm).

The 13–93 BG scaffolds were solid gray-white and printing paths of the scaffold were clearly visible (Fig. 1a–c). The hybrid scaffolds appeared as a translucent light yellow. Although both materials were printed with the same nozzles with the aim of producing scaffolds of the same dimensions, noticeable differences were observed. These variations are likely due to differences in the material shrinkage during the printing and drying processes (Table S2). The 13–93 BG scaffolds were closer to the target dimensions (8 mm diameter, 12 mm height) compared to the hybrid scaffolds. After sintering, the diameter of the 13–93 BG scaffolds measured approximately 8.9 mm, slightly exceeding the desired 8 mm with a shrinkage of 13.5% from the initial diameter (10.3 mm). The height shrinkage was moderate at about 18% (from an initial 14 mm to a final height of 11.4 mm), resulting in a final height close to the intended 12 mm. In contrast, from an initial height of 16.8 mm, the final average height of the hybrid scaffolds was approximately 9.4 mm, with a shrinkage of around 44%. This substantial reduction could also be due to the downward pressure exerted by gravity on the upper portion of the scaffold during processing. The final average diameter of the hybrid scaffolds was approximately 7.5 mm, likely due to the inner diameter of the punch was slightly smaller than the nominal 8 mm. These shrinkage values are critical for accurately predicting the final dimensions when producing 13–93 BG and hybrid material scaffolds via DIW.

## 3D imaging of 13–93 bioactive glass and hybrid material bone scaffolds

3D renderings from µCT images show that the 13–93 BG scaffolds retain a more complete cylindrical structure, whereas the hybrid scaffolds show partial structural damage because of the cutting process (Fig. 1c,d). The 13–93 BG scaffold exhibits a layered, grid-like structure with larger channel spaces. The top view of the 13–93 BG scaffold reveals more organized and dense patterns compared to the hybrid scaffolds (Fig. 1c). This highly organized framework is likely to provide high compressive strength, which is essential for load-bearing applications, and the surface of the scaffold may offer bioactivity for bonding with bone tissue. In contrast, the hybrid scaffolds exhibit an irregular structure with interconnected pores (Fig. 1d). Due to the viscosity decrease of the hybrid material during printing, gravitational settling causes the struts to compress against each other and reduces in pore size, as shown in the selected VOI of 3D hybrid scaffold rendering (Fig. 1d). This structure more mimics the natural bone matrix more closely than 13–93 BG and may provide excellent support for bone regrowth.

Both types of scaffolds were fabricated using the same DIW process, therefore their structural differences are influenced by variations in material properties, printability, and post-printing processing conditions. Prior to printing, the frozen hybrid ink was brought to room temperature to achieve an optimal viscosity that allows for smooth extrusion and stable printing. Once deposited, the ink exhibits good shape maintenance, which is advantageous for layer-by-layer stacking without collapsing. However, as printing progressed, the viscosity may decrease due to thermal exposure or shear thinning^39^, leading to increased flowability. Since our current DIW setup lacks active temperature control, it is not feasible to perform systematic rheological measurements under well-defined thermal conditions. This limitation can result in spreading or sagging after deposition, causing irregular filament formation and contributing to the development of a random, porous architecture with larger, interconnected pores^40^. In contrast, 13–93 BG mixed with Pluronic solution for printability exhibited more controlled and uniform deposition, which led to an organized structure. Post-processing further accentuates these differences: hybrid scaffolds typically undergo minimal sintering to preserve their porous architecture, while 13–93 BG scaffolds require high-temperature sintering for mechanical strength, causing densification and shrinkage that contribute to their compact structure.

Table 1 shows key microstructural parameters of the 3D printed scaffolds. The 13–93 BG scaffold exhibited higher total porosity (46%) compared to the hybrid scaffold (34%), which may enhance its ability to support bone growth and cell migration. Both material scaffolds show similarly high open porosity (> 98%), suggesting favourable osteoconductivity and the potential to facilitate tissue ingrowth. The 13–93 BG scaffold has a larger overall volume compared to the hybrid scaffold, contributing to improved mechanical stability. Its surface area (5785 mm²) is also significantly greater than that of the hybrid (1756 mm²). Additionally, the higher permeability of the 13–93 BG scaffold indicates superior fluid transport capacity, which is beneficial for nutrient delivery. These properties underscore the higher porosity, larger volume, greater surface area, and higher permeability of 13–93 BG scaffolds compared to hybrid scaffolds.

**Table 1:** Microstructural parameters of 3D-printed BG and hybrid scaffolds: porosity, open porosity, total strut volume, surface area, and permeability derived from µCT imaging.

	Porosity (%)	Percentage open porosity (%)	Total strut volume (mm^3^)	Surface area (mm^2^)	Permeability (d)
13–93 BG	46 ± 6	99.2 ± 0.1%	429 ± 29	5785 ± 373	2260 ± 168
Hybrid	34 ± 3***	98.3 ± 0.1%****	298 ± 35*	1756 ± 123***	1600 ± 319*

Statistical significance was determined using the Mann-Whitney U test: * indicates *P* < 0.05, ** indicates *P* < 0.01, *** indicates *P* < 0.001 and **** indicates *P* < 0.0001.

**Table 2:** Strut thickness and channel size of scaffolds.

	Strut thickness (µm)	Channel size (µm)
13–93 BG scaffold (*n* = 6)	301 ± 41	390 ± 124
Hybrid scaffold (*n* = 6)	363 ± 51	250 ± 92

**Fig. 2:**
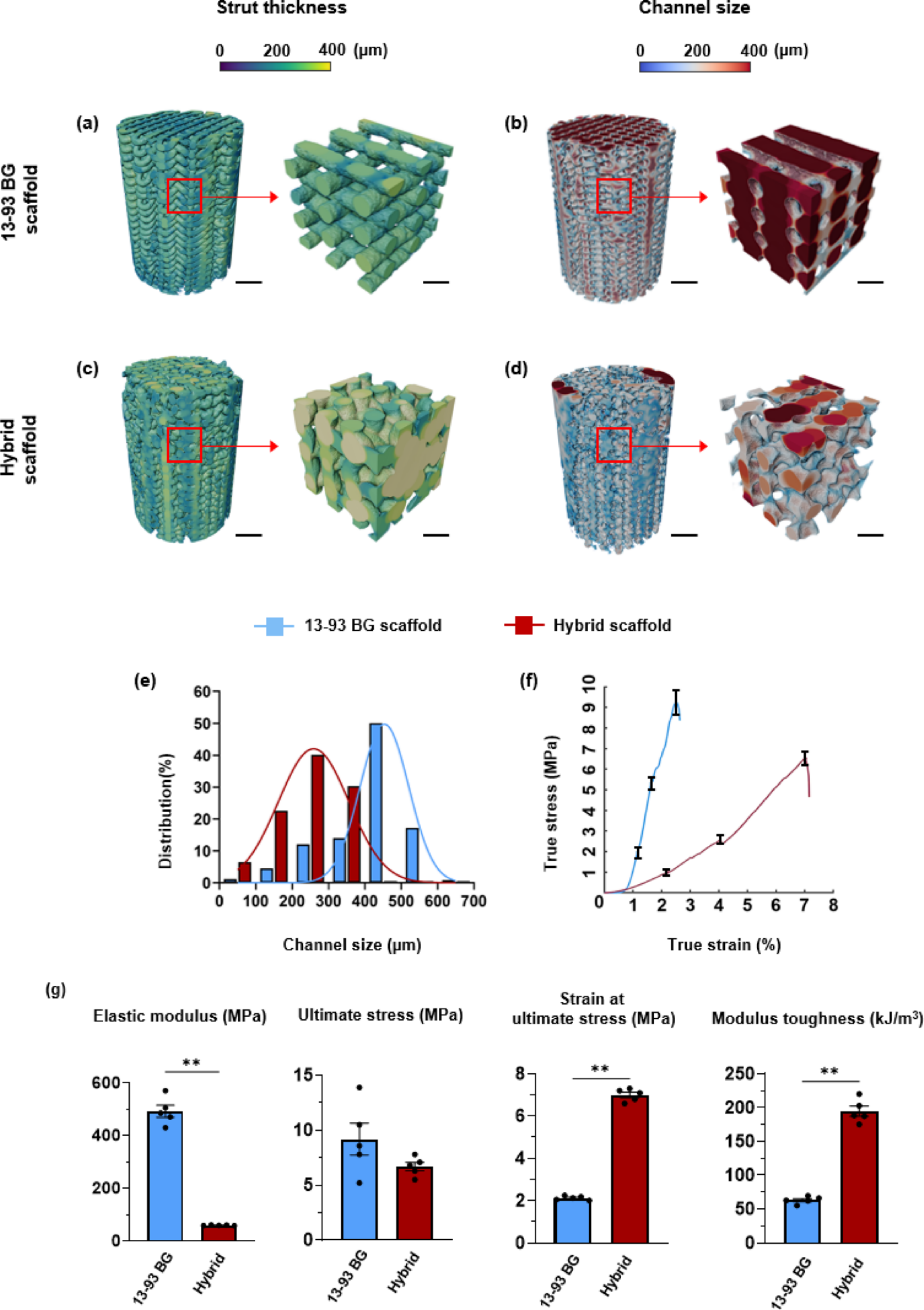
Strut thickness and channel size distribution in 3D-printed 13–93 BG and hybrid scaffolds: strut thickness colormap of (**a**) 13–93 BG and (**b**) hybrid scaffolds (scale bar 2 mm), including selected VOI (2 × 2 × 2 mm, scale bar 0.5 mm) to highlight fine structural details; channel size colormaps of (**c**) 13–93 BG and (**d**) hybrid scaffolds, with corresponding VOIs (2 × 2 × 2 mm) illustrating intricate internal features; (**e**) histograms of channel size distribution by volume fraction within the 3D-printed scaffolds; (**f**) stress–strain curves of 13–93 BG and hybrid scaffolds under uniaxial compression testing. Error bars are shown at the elastic, plastic and failure points; (**g**) mechanical parameters of 13–93 BG and hybrid scaffolds under uniaxial compression testing.

Table 2 summarizes the strut thickness and channel size. The strut thickness distribution of the 13–93 BG scaffold shows more consistent coloration, indicating a uniform strut size and a well-organized cylindrical structure (Fig. 2a). In contrast, the hybrid scaffold displays greater colour variation, reflecting a less uniform and more irregular architecture compared to the 13–93 BG scaffold (Fig. 2c). The 13–93 BG scaffold has a smaller and more uniform strut size of 301 μm compared to the hybrid scaffold, which averages 362 μm. These differences in strut thickness may influence mechanical properties such as the elastic modulus, suggesting that the two materials will perform differently in bone regeneration^41,42^. Figure 2b and d show the channel size distributions of the 13–93 BG and hybrid scaffolds. The hybrid scaffold had an average channel size of 250 μm, while the 13–93 BG scaffold shows a larger average pore size of 390 μm with a more homogeneous pore architecture. In the selected VOI, the channel size distribution of the 13–93 BG scaffold reveals widespread dark red regions along the vertical struts and pore walls, indicating a consistent presence of larger pores exceeding 300 μm. In contrast, the hybrid scaffold exhibits a more irregular and disordered pore network with dominant shades of blue and grey in the selected VOI, representing smaller and more variable pores below the 300 μm threshold. These differences in channel size and distribution are likely to influence each scaffold’s ability to support nutrient diffusion and cell migration, which are critical for successful bone regeneration.

The minimum pore size required for in vivo bone ingrowth is generally accepted to be 100 μm^43^. Both 13–93 BG and hybrid scaffolds exceed this threshold, highlighting their potential as effective candidates for supporting bone regeneration. The channel size distribution histogram shows that the 13–93 BG scaffolds exhibit a larger average channel size with a distinct peak between 400 and 500 μm (Fig. 2e). The dominance of larger pores may support vascularisation and nutrient diffusion, which are essential in early-stage bone healing^44^. However, the reduced strut thickness and large channel size may compromise its mechanical strength of 13–93 BG. The enhanced vascularisation potential of the 13–93 BG scaffolds could make them ideal for applications requiring rapid development of a robust vascular network, such as during early fracture healing^45^. The hybrid scaffolds exhibit channel sizes predominantly in the 200–300 μm range. This pore structure results from thicker struts and shrinkage effects during processing, which create a more disordered pore network despite the lower measured total porosity. This architecture may offer an ideal environment for progressive bone ingrowth over time, as large pores (200–400 μm) facilitate nutrient diffusion, vascularisation, and bone tissue ingrowth^44,46^. This variation in pore size distribution between the two materials highlights their differing strengths and potential applications in bone tissue engineering.

Shi et al. demonstrated that the ICIE16 melt-derived bioactive glass, designed with 250 μm struts and 160 μm channels undergoes controlled viscous sintering, yielding final features of 166.9 ± 19.4 μm (strut thickness) and 153.3 ± 6.4 μm (channel size), corresponding to 33.2% strut shrinkage but only 4.2% channel reduction^26^. This near-isotropic densification and minimal pore collapse, coupled with a porosity of 46.4 ± 3.3%, reflect the well-established sintering behaviour of low-viscosity silicate glasses above their glass transition temperature (600–700 °C). By contrast, our 13–93 BG scaffolds designed with larger initial features (332 μm struts, 650 μm channels), they undergo only modest strut shrinkage (9.3%) yet suffer severe channel collapse (40.0%), resulting in final dimensions of 301 ± 41 μm (strut) and 390 ± 124 μm (channel). achieving a comparable porosity (46 ± 6%). In conclusion, while both systems achieve similar final porosities, the ICIE16 glass exhibits superior structural fidelity with uniform densification, whereas the 13–93 BG scaffolds display anisotropic deformation characterized by disproportionate channel collapse despite larger initial dimensions.

Similarly, the calcium-free TEOS/PCL-diCOOH (80:20) scaffold, designed with a strut thickness of 200 μm, exhibits approximately 35% channel reduction (from 200 μm to 130 μm) with a porosity of 42%^21^. The interconnected channel size ranged from 40 to 240 μm and strut diameters ranged between 140 and 200 μm. In contrast, our calcium-containing hybrid scaffold ink exhibits a minimal strut shrinkage (9.3%) and suffers 37.5% channel reduction (from 400 μm to 250 μm). Compared to our calcium hybrid scaffolds, the adding of calcium results in improved print stability of the struts, while maintaining comparable channel reduction. Notably, despite the higher initial designed channel size (400 μm vs. 200 μm), the final channel dimension in our scaffold remains larger (250 μm vs. 130 μm), which may be advantageous for enhancing pore interconnectivity and facilitating cell infiltration and vascularisation.

## Uniaxial compressive testing

The stress–strain curve of the 13–93 BG scaffolds illustrates their mechanical response under compression (Fig. 2f). In the initial elastic region, a steep increase in stress reflects their high stiffness, attributed to the strong, rigid Si–O and Ca–O bonds within the amorphous silica network^47,48^ The curve reaches a maximum stress of 9.2 MPa, representing the sxaffolds’ maximum load-bearing capacity. However, the brittle nature of Si–O bonds^49^ and the lack of slip systems for plastic deformation lead to rapid bond failure and minimal yielding^50^. Abrupt failure highlights the inherent brittleness of 13–93 BG and its limited ability to resist crack growth. In contrast, the stress–strain curve of the hybrid scaffold (Fig. 2f) shows an initial elastic region that reflects its lower stiffness, influenced by a combination of covalent bonds (e.g., Si–O, C–O) and weaker ionic interactions (e.g., Ca–O). Beyond this region, the curve transitions into a nonlinear phase before reaching a peak stress of 6.7 MPa. Compared to the 13–93 BG scaffold, the hybrid shows greater strain at peak stress, likely due to molecular level interactions between the polymer matrix and the inorganic silica phase. After reaching its peak, the hybrid scaffold also experiences a sharp decline in stress due to microstructural collapse.

The key mechanical parameters of the hybrid and 13–93 BG scaffolds are presented in Fig. 2g and Table S3. Human trabecular bone typically exhibits an elastic modulus ranging from 800 to 2700 MPa^51^, which is higher than that of the 13–93 BG scaffold (492 MPa). In contrast, the hybrid scaffold shows a much lower average elastic modulus of 59.7 MPa, indicating reduced stiffness relative to both 13–93 BG and native trabecular bone. Trabecular bone generally has an ultimate compressive strength between 2 and 12 MPa^24^ and a corresponding strain at ultimate stress ranging from 10% to 30%^52^. The hybrid scaffold’s ultimate stress of 6.7 MPa is within this range but is lower than that of the 13–93 BG (9.2 MPa), suggesting slightly reduced peak strength. However, the hybrid demonstrates a higher strain at failure (7%) compared to 13–93 BG (2%), indicating greater deformability and a response more similar to native bone. Moreover, the modulus of toughness of the hybrid scaffold is 193.6 kJ/m^3^, approximately three times greater than that of 13–93 BG (63.8 kJ/m^3^). This enhanced toughness reflects a superior capacity for energy absorption, allowing the material to better dissipate mechanical stress. Nonetheless, the relatively low elastic modulus of the hybrid scaffold suggests that further optimization is needed to achieve full biomechanical compatibility with native bone. In general, hybrid scaffolds are well-suited for applications requiring energy absorption, deformability, and resistance to progressive collapse, making them ideal for dynamic or cyclic loading scenarios, such as bone regeneration in the knee^53,54^. In contrast, 13–93 BG scaffolds are optimized for applications demanding high initial stiffness and load-bearing capacity under static conditions. They are particularly advantageous as components in composite scaffolds, where their stiffness can be balanced by incorporating a ductile phase to mitigate brittleness.

Cyclic uniaxial compression testing was performed on 3D printed hybrid bone scaffolds to assess mechanical stability under repeated loading (Fig. S2). The stress-strain response over 10 loading cycles is shown, with magnified views of the 1st and 10th cycles at high stress range. Under a maximum applied load of 50 N, the average peak true stress reached approximately 1.25 MPa, corresponding to a maximum true strain of 4.8%. Notable differences were observed between the 1st and 10th cycles. In the 1st cycle, peak stress was achieved at 4.5% strain, with a relatively shallow initial slope and irregular fluctuations in the elastic region. These initial variations are most likely due to non-uniformities in the cylindrical geometry and internal porosity of the scaffold, where localized collapse or microfractures at the top surface may have occurred during initial loading. From the 4th cycle onward, the scaffold exhibited a smoother and more uniform loading response, reaching a slightly higher peak stress at a similar strain (~ 4.8%). This improvement suggests that compaction and surface flattening during early cycles contributed to mechanical stabilization. The presence of hysteresis loops, which progressively narrow over the 10 cycles, indicates decreasing energy dissipation and improved elastic recovery. The shift in peak strain to higher values further reflects cyclic adaptation, as the scaffold becomes more resilient with repeated loading^55^. This mechanical conditioning behaviour may be advantageous in vivo, where the scaffold must endure repetitive physiological stresses while maintaining structural integrity^23^.

### Finite element simulation of uniaxial compression testing

**Fig. 3:**
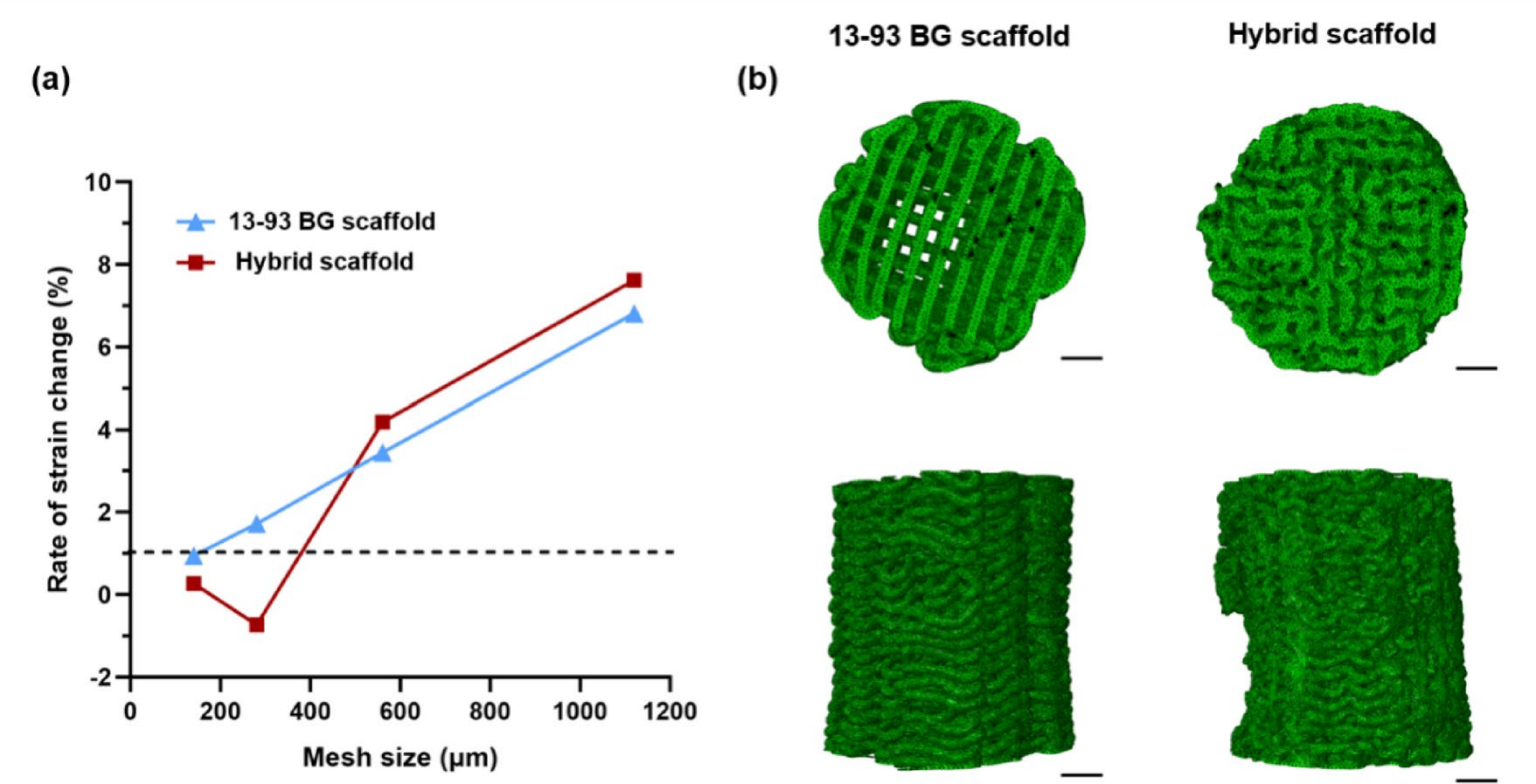
FE model element size selection and meshing used in the simulation of 13–93 BG and hybrid scaffolds: (**a**) mesh convergence analysis of scaffold models showing the variation in first principal strain with decreasing mesh size. The horizontal dashed line at 1% indicates the convergence threshold; (**b**) representative images of meshed 13–93 BG and hybrid scaffold models used in the simulations (scale bar 1.5 mm).

In the mesh convergence analysis, a strain difference of less than 1% was considered indicative of convergence. A mesh size of 140 μm was selected for both the 13–93 BG and hybrid scaffold models to achieve a balance between FE accuracy and computational efficiency (Fig. 3a). Details of the meshed models for the 13–93 bioactive glass and hybrid scaffolds are shown in Fig. 3b. The top and bottom surfaces are flat and the difference in overall geometry between the two scaffold types is clearly visible. The meshed models capture most of the 3D-printed scaffold geometries including small features near the edges, which were preserved by employing a smaller element size.

**Fig. 4:**
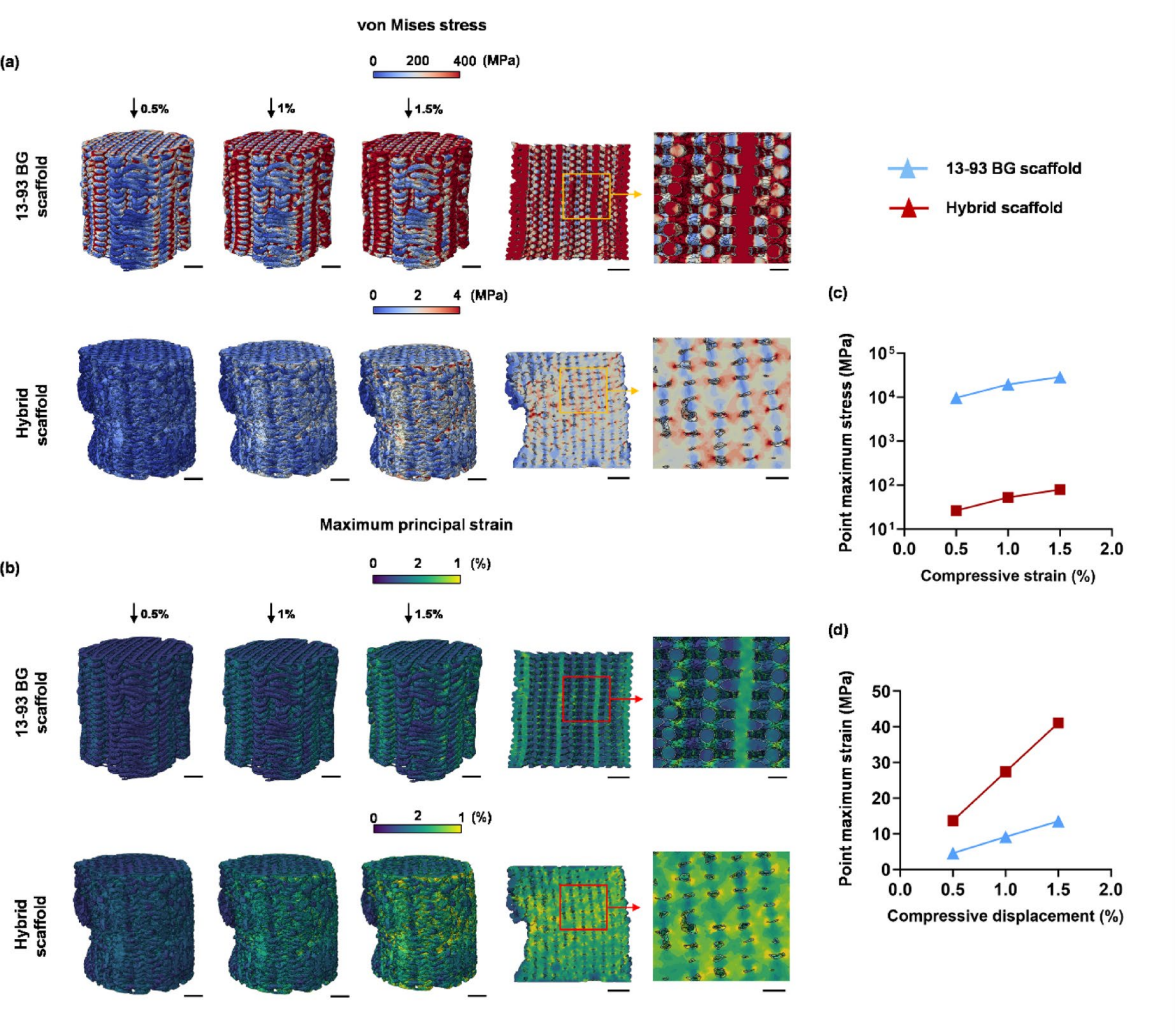
FE prediction of local stress and strain distributions in 13–93 BG and hybrid scaffolds under nominal compressive strains of 0.5%, 1.0%, and 1.5%: distribution and magnitude of predicted (**a**) von Mises stress and (**b**) principal strain (scale bar: 1.5 mm); predicted point maximum (**c**) von Mises stress and (**d**) principal strain at 0.5%, 1.0%, and 1.5% engineering compressive strain. Note the peak strain values represent highly localized strain concentrations within a small number of elements in the scaffold microarchitecture and should not be interpreted as macroscopic strain capacity or large-deformation behaviour of the scaffold.

The 13–93 BG and hybrid scaffold models were subjected to uniaxial compression simulations at displacements of 0.45 mm (0.5%), 0.90 mm (1%), and 1.35 mm (1.5%) relative to the original 9 mm height. Figure 4 presents the results of the FE analysis, showing the stress and strain distributions for both scaffold models. With increasing displacement, both stress and strain values rise in the 13–93 BG and hybrid scaffold models, reflecting progressive deformation and compression of the structures (Fig. 4a,b). The von Mises stress (overall stress state within element) was much higher in the 13–93 BG model compared to the hybrid model. Section views of the stress distribution model for the 13–93 BG scaffold revealed that stress concentrations were primarily located at the junctions between struts. As compressive displacement increased, these high-stress regions progressively expanded outward from the connection points with most stress values exceeding 300 MPa. In contrast, the hybrid scaffold exhibited a more uniform and randomly distributed stress pattern with overall lower stress magnitudes (< 4 MPa). These results suggest that the hybrid scaffold may offer improved stress distribution and potentially better mechanical resilience under compressive loading. The strain distribution patterns of the two scaffold models exhibited similar overall spatial trends to their stress distributions. However, the magnitude of strain within the hybrid scaffold was much higher than that within 13–93 BG scaffold. Strain was predominantly concentrated at the junctions between struts in the 13–93 BG model, whereas strain was more diffusely and randomly distributed throughout the structure in the hybrid model. Moreover, the overall strain values in the 13–93 BG scaffold were relatively low, while the hybrid scaffold demonstrated substantially higher strain levels.

It should be noted that these simulations were based on a linear-elastic constitutive description and are therefore most appropriate for comparative analysis within the initial elastic regime. Accordingly, the high local principal strain values predicted in the hybrid scaffold should be understood as highly localised strain concentrations within geometrically constrained regions of the porous architecture, rather than as evidence of macroscopic strain capacity or physically realistic large-strain constitutive behaviour. More accurate prediction of large-deformation and failure responses, especially in the polymer-containing hybrid scaffold, would require nonlinear material models together with improved contact definitions. The FE simulations were designed to assess relative micromechanical behaviour and strain localization patterns rather than to extract quantitatively accurate peak stress values. Therefore, the extreme local stress and strain values reported here should be interpreted as indicators of damage-prone regions rather than as physically representative stresses of the bulk scaffold material. These FE results were designed to provide comparative insight into the micromechanical response of the two materials scaffolds under small-strain elastic conditions, rather than to deliver quantitative predictions of stress–strain curves, failure behaviour, or behaviour in the large-deformation region.

Figure 4c and d show the predicted point maximum stress and strain values of the 13–93 BG and hybrid scaffold models. The hybrid scaffold also showed a strong linear increase in strain with compressive displacement and reached over 40% localized strain (only occurred within a very small number of elements) at 1.5% displacement. The 13–93 BG scaffold had much lower strain values, increasing from around 5% to 13%. Overall, the 13–93 BG scaffold model shows much higher compressive strength but low strain tolerance, which indicate a brittle behaviour. The hybrid scaffold model had a much higher strain capacity, suggesting better ductility and energy absorption, though at lower stress levels. These results suggest that the hybrid scaffold possesses greater deformability and ductility under compression. This may allow it to better handle mechanical loads without causing localized failure. In contrast, the 13–93 BG scaffold’s lower point strain and more focused distribution may indicate a higher brittleness and a greater risk of fracture initiation at the strut junctions.

## Conclusion

This study compared 13–93 BG and hybrid scaffolds. 3D printed by direct ink writing using the same design file. Although the two scaffold systems differ in composition, both were designed for the same load-bearing bone repair application and were fabricated and tested using the same direct ink writing and mechanical characterisation framework. This allowed the difference between the two material systems in terms of scaffold microstructure, deformation behaviour, and compressive performance to be examined under comparable processing and loading conditions.

The hybrid material showed lower density and elastic modulus compared to the 13–93 BG material. The differing printability of the two materials was attributed to variations in their ink properties. Printing the 13–93 BG scaffold was easier to control because of the Pluronic-based carrier. In contrast, the hybrid material was more difficult to print due to viscosity changes during the printing process. This challenge could be addressed by modifying the material composition or using temperature-controlled 3D printers to maintain viscosity. µCT analysis confirmed that both scaffolds exhibited a well-defined porous architecture with an interconnected average channel size exceeding 200 μm, which is essential for cellular infiltration and bone ingrowth. The 13–93 BG scaffolds displayed a more regular and less dense pattern, whereas the hybrid material scaffolds displayed an irregular, bone-matrix-like structure with smaller, heterogeneous channels. Mechanical testing showed that the hybrid scaffold had three times greater toughness, higher deformability (7% strain), and better elastic recovery under cyclic loading. In contrast, the 13–93 BG scaffold had a higher elastic modulus but was limited by brittleness. FE analysis supported these results that the 13–93 scaffold showed high stress and low strain with localized stress concentration. The hybrid scaffold showed lower stress and higher strain with a more diffuse distribution, showing greater ductility and deformability under compression. However, predicting large strain and failure behaviour accurately requires more refined material properties, improved boundary conditions, and FE models capable of simulating compression until failure. Also, future studies should seek to incorporate explicit platen contact and friction to more closely replicate experimental conditions. While these findings highlight the structural and mechanical advantages of hybrid scaffolds, further studies are necessary to assess their long-term degradation behaviour, cellular response and in vivo performance. Future research should include SEM/EDS analysis to capture high-resolution microstructural images and elemental maps both before and after compression testing, offering valuable insights into deformation behaviour, structural integrity, and potential load-induced compositional changes.

Concurrently, work should focus on optimizing scaffold composition, enhancing biocompatibility, and conducting rigorous preclinical evaluations to validate the efficacy of these biomaterials in promoting bone healing. This study establishes a comprehensive and reproducible characterisation protocol that integrates quantitative mechanical testing with high-resolution 3D imaging modalities, enabling an advanced, approach to the evaluation of biomaterial implants. The contribution of this work extends beyond a material-to-material comparison. By combining micro-CT-based structural analysis, experimental mechanical testing, and finite element modelling on the same scaffold systems, a transferable framework for linking architecture, local mechanical response, and bulk performance was established. This methodology is not limited to hybrid and 13–93 bioactive glass scaffolds, but can also be applied more broadly to the design, optimization, and evaluation of the development, optimization, and regulatory assessment of next-generation biomaterials for bone regeneration.

## Electronic Supplementary Material

Below is the link to the electronic supplementary material.

Supplementary Material 1

## Data Availability

All data supporting the findings of this study are available within the paper and its supplementary information.
