# Supplementary material for: Comparative mechanical characterisation of 13–93 bioactive glass and hybrid scaffolds for bone regeneration
Source: Sci Rep. 2026 Apr 3;16:15905. doi: 10.1038/s41598-026-46620-9 (PMC13194938; doi:10.1038/s41598-026-46620-9)
Supplement: Supplementary file 1 — Supplementary Material 1 [file 41598_2026_46620_MOESM1_ESM.docx]

# **Supplementary Information**


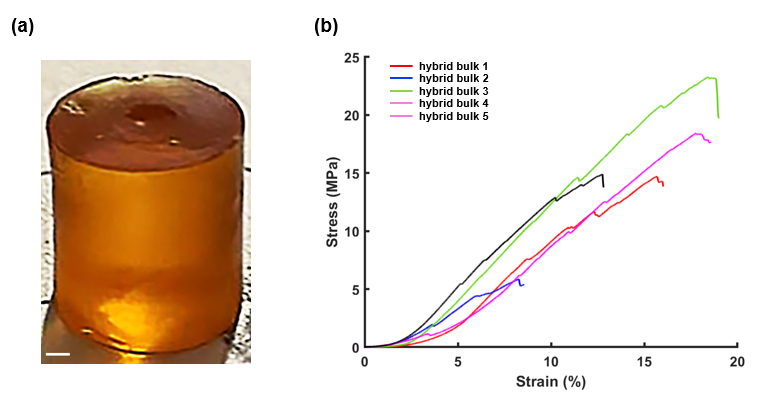


**Figure S1.** Material properties of hybrid bulk samples: (a) representative images of bulk samples (scale bar 1 mm); (b) Stress-strain curve illustrating the mechanical performance of hybrid bulk samples under uniaxial compression.


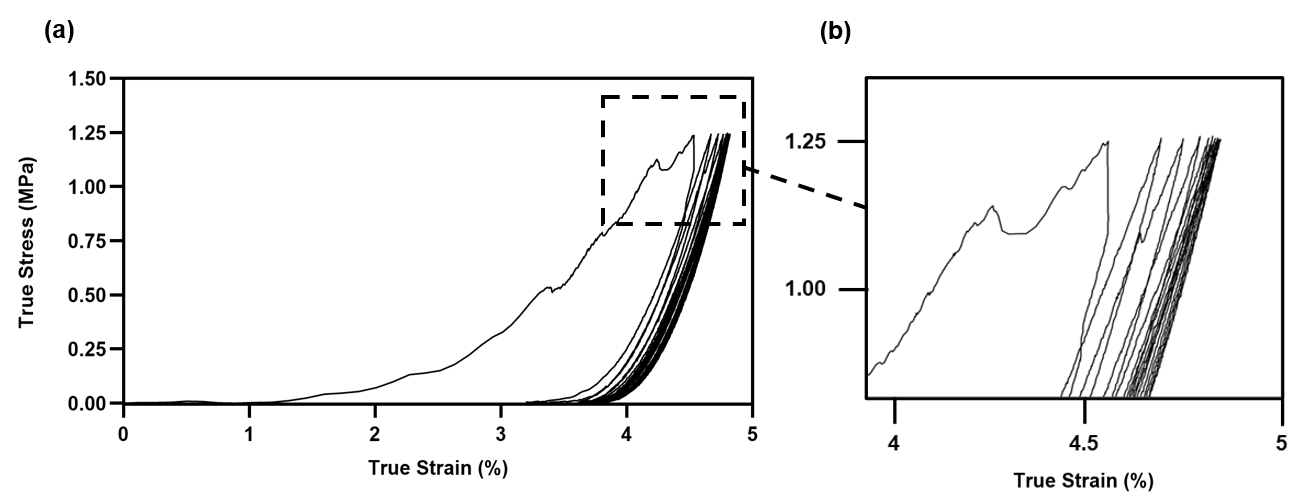


**Figure S2.** True stress–strain curves of 3D-printed hybrid bone scaffolds under cyclic uniaxial compressive loading: (a) Stress–strain response over 10 loading cycles; (b) Enlarged view of cycles 1 to 10, highlighting the progressive changes in mechanical behaviour with repeated loading.

|  | **Density**  **(kg/m³)** | **Elastic Modulus (MPa)** | **Modulus Toughness**  **(kJ/m^3^)** | **Compressive strength (MPa)** |
| --- | --- | --- | --- | --- |
| 13-93 BG | 2440-2880 | 86000 | -- | 69 |
| Hybrid | 1370 | 139 ± 16 | 1395 ± 550 | 18 ± 4 |

**Table S1.** Mechanical properties of the tested 13-93 bioactive glass and hybrid material: density, elastic modulus, toughness, and compressive strength.

| **Sample** | **13-93 BG scaffolds** | **Hybrid bone scaffolds** |
| --- | --- | --- |
| Printing dimension | Height: 14 mm  Diameter: 10.3 mm | Height: 16.8 mm  Diameter: 8 mm |
| Dimension | Height: 11.4 ± 0.6 mm  Diameter: 8.9 ± 0.3 mm | Height: 9.4 ± 0.1 mm  Diameter: 7.5 ± 0.4 mm |
| Shrinkage/difference | Height: 13.5 ± 0.1%  Diameter: 18 ± 0.1% | Height: 44 ± 0. %  Diameter: 0.5 ± 0.4 mm |

**Table S2.** Dimension and shrinkage of printed scaffolds.

|  | **Compressive Modulus (MPa)** | **Ultimate Stress (MPa)** | **Strain at Ultimate Stress** | **Modulus Toughness (kJ/m^3)** |
| --- | --- | --- | --- | --- |
| 1393 BG scaffold (n=6) | 492.1 ± 21.5 | 9.2 ± 2.3 | 2 ± 0.4% | 63.8 ± 3.1 |
| Hybrid scaffold (n=6) | 59.7 ± 7.0 | 6.7 ± 1.7 | 7 ± 0.8% | 193.6 ± 12.4 |
| Human trabecular bone | 800 - 2700 | 2 - 12 | 10 - 30%. | / |

**Table S3.** Mechanical properties of bone scaffolds and human trabecular bone

| **Channel Size (µm)** | **Hybrid scaffold** | **13-93 BG scaffold** |
| --- | --- | --- |
| 0-100 | 6.5% | 1.2% |
| 100-200 | 22.5% | 4.5% |
| 200-300 | 40.1% | 12.1% |
| 300-400 | 30.3% | 14.0% |
| 400-500 | 0.5% | 50.0% |
| 500-600 | 0% | 17.2% |
| 600-700 | 0% | 0.9% |

**Table S4** Channel size distribution of hybrid and 13-93 BG scaffolds
